# Impact of clinical presentation and presence of coronary sclerosis on long-term outcome of patients with non-obstructive coronary artery disease

**DOI:** 10.1186/s12872-018-0908-z

**Published:** 2018-08-22

**Authors:** Christine K. Kissel, Guanmin Chen, Danielle A. Southern, P. Diane Galbraith, Todd J. Anderson, for the APPROACH investigators

**Affiliations:** 1 Department of Cardiac Sciences and Libin Cardiovascular Institute of Alberta, Cumming School of Medicine, University of Calgary, Calgary, AB Canada; 2 Department of Cardiology, University Heart Center, University Hospital Zurich, Rämistrasse 100, CH-8091 Zürich, Switzerland; 3 O’Brien Institute of Public Health, Cumming School of Medicine, University of Calgary, Calgary, AB Canada; 4 Department of Community Health Sciences, Cumming School of Medicine, University of Calgary, Calgary, Canada

**Keywords:** Coronary artery disease, Prognosis, Stable angina, Acute coronary syndrome

## Abstract

**Background:**

Non-obstructive coronary artery disease (NOCAD) is a common finding on coronary angiography. Our goal was to evaluate the long-term prognosis of NOCAD patients with stable angina (SA).

**Methods:**

The study cohort consisted of 7478 NOCAD patients with normal EF (≥ 50%), and SA who underwent coronary angiography between 1995 and 2012. We compared NOCAD patients (stenosis< 50%) with 10,906 patients with stable obstructive CAD (≥ 50%). The primary endpoint was all-cause mortality. Secondary endpoints included repeat angiography, progressive CAD, and PCI. A second comparison group consisted of 7344 patients with NOCAD presenting with an ACS. Rates of all-cause mortality of NOCAD ACS patients were compared to NOCAD SA patients.

**Results:**

Median follow-up time was 6.5 years. NOCAD patients had a lower risk of all-cause mortality compared to CAD patients (HR CAD vs. NOCAD 1.33 (1.19–1.49); *p* < 0.001). This was driven by patients with normal coronary arteries (HR CAD vs. normal 1.63 (1.36–1.94), *p* < 0.001), whereas patients with minimal disease (> 0% and < 50%) were at similar risk as CAD patients (HR CAD vs. minimal 1.08 (0.99–1.29), *p* = 0.06). In NOCAD patients, the strongest predictors of all-cause mortality were age and minimal disease. SA patients with NOCAD had low rates of repeat angiography (7.3%), future CAD (2.3%) and PCI (1.7%). NOCAD ACS patients had a 41% increase in all-cause mortality risk compared to NOCAD SA patients (HR 1.41 (1.25–1.6), *p* < 0.001).

**Conclusions:**

This study underlines the importance of minimal CAD, as it is not a benign disease entity and portends a similar risk as stable obstructive CAD.

**Electronic supplementary material:**

The online version of this article (10.1186/s12872-018-0908-z) contains supplementary material, which is available to authorized users.

## Background

Non-obstructive coronary artery disease (NOCAD) is a common finding on diagnostic coronary angiograms with rates of up to 50–60% in patients with stable angina (SA) and of about 30% in certain population with acute coronary syndromes (ACS) [1–4]. Symptomatic patients with NOCAD have often been reassured of the innocuousness of the results, and frequently no further preventive measures were taken [5, 6]. The etiology of symptoms in these patients appears to be heterogeneous and prognosis was often deemed favourable. Recently, the conception that NOCAD is a benign disease has been challenged [3, 4, 6, 7]. Jespersen et al. showed a graded increase in major adverse cardiovascular events (MACE) in patients with normal arteries, non-obstructive, and obstructive coronary disease. These findings were confirmed by Maddox et al. who demonstrated an increase of all-cause mortality and MI rate from non-obstructive to obstructive CAD by extent of vessel distribution in a cohort of mostly male veterans presenting for an elective coronary angiogram [7]. One of the first study groups who followed NOCAD patients systematically was the WISE study group (Women’s Ischemia Syndrome Evaluation). WISE primarily examined women, who were found to have cardiac syndrome X, i.e. chest pain of an ischemic origin and NOCAD. The authors demonstrated a high rate of all-cause mortality rate (18% in 10 years), and high rates of repeat angiogram (19%) in women with NOCAD and SA [3]. In contrast, the Swedish Coronary Angiography and Angioplasty Registry (SCAAR) registry demonstrated a low all-cause mortality of 0.3–0.4% in NOCAD patients with SA at 2 years [2], hereby raising the question about contemporary, long-term all-cause mortality in NOCAD.

Furthermore, it is well known that presentation with an ACS with obstructive CAD portends an increased mortality risk. Previous studies addressed ACS patients with NOCAD, which is also termed myocardial infarction with non-obstructive coronary arteries (MINOCA), but study size was either small or patients were followed only in the short-term [8–12].

Our aim was therefore to evaluate prognosis and its predictors in a large, contemporary population of patients of both sexes with NOCAD. We also sought to investigate whether presentation with an ACS leads to a worse prognosis compared to stable NOCAD patients.

## Methods

### Data source and collection

Eligible subjects included all adults over the age of 18 years undergoing their first cardiac catheterization between January 1, 1995 to March 31, 2012, registered in the Alberta Provincial Project for Outcome Assessment in Coronary Heart Disease (APPROACH©) database [13]. APPROACH is a prospective cohort of all adults undergoing cardiac catheterization in Alberta, Canada. APPROACH contains detailed patient information, as well as specifics on coronary anatomy and therapeutic interventions. Data were entered at time of catheterization and are routinely enhanced by merging the clinical registry data to administrative records.

Data collection included e.g. sociodemographic characteristics, comorbidities and risk factors, disease specific variables (e.g. indication for procedure, angina status), and medications at time of catheterization. Angiography results including coronary anatomy, extent of coronary stenosis, and LV ejection fraction (EF) were also recorded [13]. The degree of stenosis was visually assessed by the angiographer with no quantitative measurement. Subsequent angiographies and revascularization procedures are also collected.

APPROACH and this protocol were approved in accordance with the Declaration of Helsinki by the Institutional Ethics Review Board of the University of Calgary. Patients signed informed consent to allow data collection, clinical follow-up, and anonymous data reporting.

### Study population

Normal coronaries, minimal disease (i.e. coronary sclerosis) and significant obstructive disease were defined as 0%, > 0 and < 50% and ≥ 50% luminal narrowing in any epicardial coronary artery, respectively. The NOCAD cohort consisted of those with normal coronary arteries or minimal disease. We identified patients with a normal EF (≥50%) who presented with either SA or an ACS.

Stable angina (SA) was defined as Canadian Cardiovascular Society (CCS) class 1, 2 or 3 angina with inclusion of clinically stable patients with atypical chest pain. ACS was defined as unstable angina or non–ST-elevation MI or ST-elevation MI in accordance to universal myocardial infarction criteria.

Patients with an EF below 50% or prior percutaneous coronary intervention (PCI) or coronary artery bypass grafting (CABG) were excluded, as well as patients with significant valvular disease, or left main disease, or referrals for pre- or post-transplant work-up, evaluation of heart failure, congenital heart disease, or serious arrhythmia. Patients with incomplete data were likewise excluded. The rate of incomplete data was low (3.7%).

The main study group consisted of patients with NOCAD (coronary stenosis < 50%) presenting with SA (*n* = 7478). Comparison Group I contained patients with significant CAD (≥ 50% stenosis) who presented with SA (*n* = 10,906). Comparison Group II included patients with NOCAD presenting with an ACS (*n* = 7344).

### Outcomes

The primary end-point was all-cause mortality with the primary efficacy analysis consisting of a comparison between CAD and NOCAD patients with SA. Follow-up all-cause mortality was ascertained through semi-annual linkage to the Alberta Bureau of Vital Statistics. The survival time from the date of first catheterization was calculated using the date of death. The survival time was censored if the patient was still alive on 31 March 2012. Secondary endpoints were development of obstructive CAD (≥ 50% stenosis on subsequent angiograms), repeat angiogram, and future PCI in NOCAD patients. Any patient who had a second angiogram during the follow-up period was counted once as having had a repeat angiogram.

### Statistical methods

Summary statistics for categorical variables presented include counts and percentage, and for continuous variables include mean and standard deviation. The difference for categorical variables between NOCAD and CAD was tested by Chi-square test; and the difference for continuous variables were evaluated by student’s t-test. Cox’s proportional hazard regression model was used to compare all-cause mortality between CAD and NOCAD. The crude hazard ratio (HR) and adjusted HR with their 95% confidence intervals were estimated. In the multivariable-adjusted model, we adjusted the HRs for age, diabetes mellitus (DM), and hypertension. The analyses for Cox’s proportional hazard regression model were stratified by the groups of NOCAD and its subgroups of normal coronaries, and minimal disease (> 0 and < 50%), as well as obstructive CAD. We estimated HRs between NOCAD and CAD patients, stratified by coronary status, and between NOCAD patients presenting with SA or an ACS.

Moreover, Cox’s proportional hazard regression models were used to investigate predictors for all-cause mortality among patients with NOCAD. Pre-specified variables for the univariate Cox regression model included age over 55 years, presence of DM, positive stress test, abnormal baseline ECG, hypertension, or previous or current smoking, as well as having undergone more than two angiograms for the same condition.

Factors, which showed a significant association with increased mortality in the univariate analysis, were entered in the multivariate regression model using a stepwise method. To explore the effect of sex, we included this factor in our analyses, independently of the significance level in the univariate model. We conducted these analyses for NOCAD patients with SA. All statistical analyses were performed using statistical software SAS (Version 9.3, Institute Inc., Cary, NC).

## Results

**Fig. 1:**
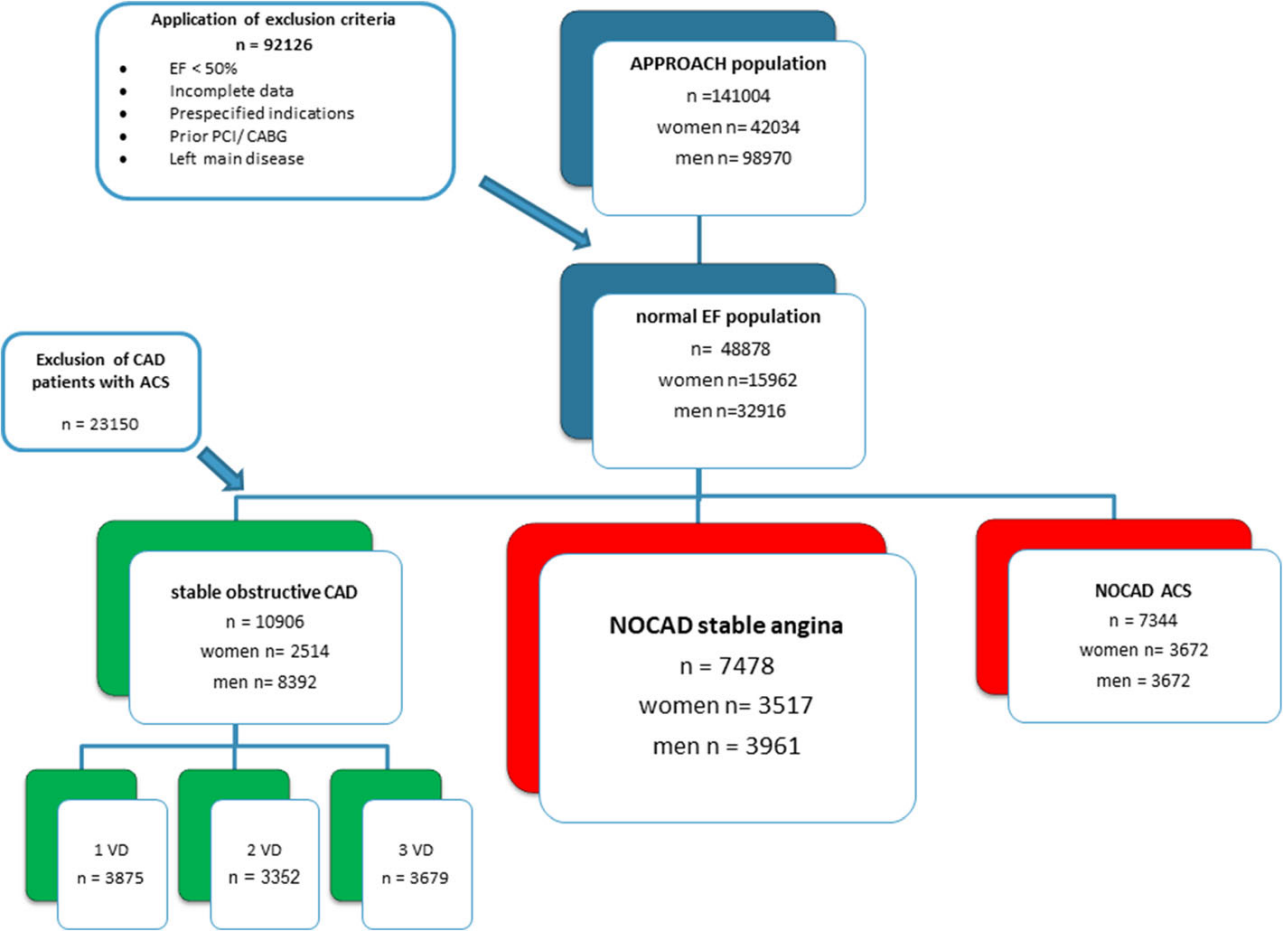
Flow diagram of patient population

During the study period, there were 141,004 patients in the APPROACH registry with 70.2% men and 29.8% women. For reasons previously described, we excluded 92,126 patients, leaving a population of 48,878 subjects with normal EF. Figure 1 shows that of the normal EF group, 7478 subjects had NOCAD presenting with SA. In the NOCAD subgroup, the percentage of women was substantially higher than the total APPROACH population (48.5%). NOCAD was found in 40.7% of all patients presenting with SA, whereas the rate for NOCAD in patients presenting with an ACS was 24.1% (Fig. 1). The median follow-up time was 6.5 years, the maximum was 13.5 years.

### Baseline characteristics of NOCAD population with SA

NOCAD patients were significantly younger, were more likely to be female and had significantly lower rates of cardiovascular risk factors compared to CAD patients. A similar picture emerged when patients with normal coronary arteries were compared to patients with minimal disease (Additional file 1: Table S1). At the time of catheterization, medication use was higher in the CAD population (Table 1).

**Table 1:** Comparison of Baseline Characteristics of Stable Angina Patients: NOCAD versus CAD

	NOCAD (*n* = 7478)	CAD (*n* = 10,906)	*p*-value*
Age, mean years	58.8 ± 10.9	64.0 ± 10.2	< 0.001
Female (%)	3517 (47%)	2514 (23.1%)	< 0.001
EF calculated (*n* = 2599)	64.9 ± 7.5	63.7 ± 12.6	< 0.001
Normal coronary arteries	49.4%	N/A	N/A
Cardiovascular risk factors:			
Hypertension (%)	60.4	72.1	< 0.001
Dyslipidemia (%)	65.6	79.2	< 0.001
Diabetes mellitus (%)	16.8	26.6	< 0.001
Smoker- current/ previous (%)	52.5	61.6	0.04
Current smoker (%)	17.7	18.9	< 0.001
Positive family history (%)	29.6	29.1	0.47
Medications at time of cath:			
Aspirin	5286/7159 (73.8%)	8940/10555 (84.7%)	< 0.001
P2Y12 Inhibitor	480/6878 (7%)	1105/10078 (11.0%)	< 0.001
Beta-blockers	3561/7060 (50.4%)	6626/10423 (63.6%)	< 0.001
Statins	3285/6926 (47.4%)	6681/10048 (66.5%)	< 0.001
Calcium channel blockers	1269/6911 (18.4%)	2343/10115 (23.2%)	< 0.001
ACE-inhibitor	1957/6969 (28.1%)	3983/10429 (38.2%)	< 0.001
Long acting nitrates	867/6862 (12.6%)	1716/ 10,043 (7.1%)	< 0.001
Insulin	193/5760 (3.4%)	445/8442 (5.3%)	< 0.001

*for comparison NOCAD vs. CAD

### Comparison of all-cause mortality of NOCAD versus CAD patients

**Fig. 2:**
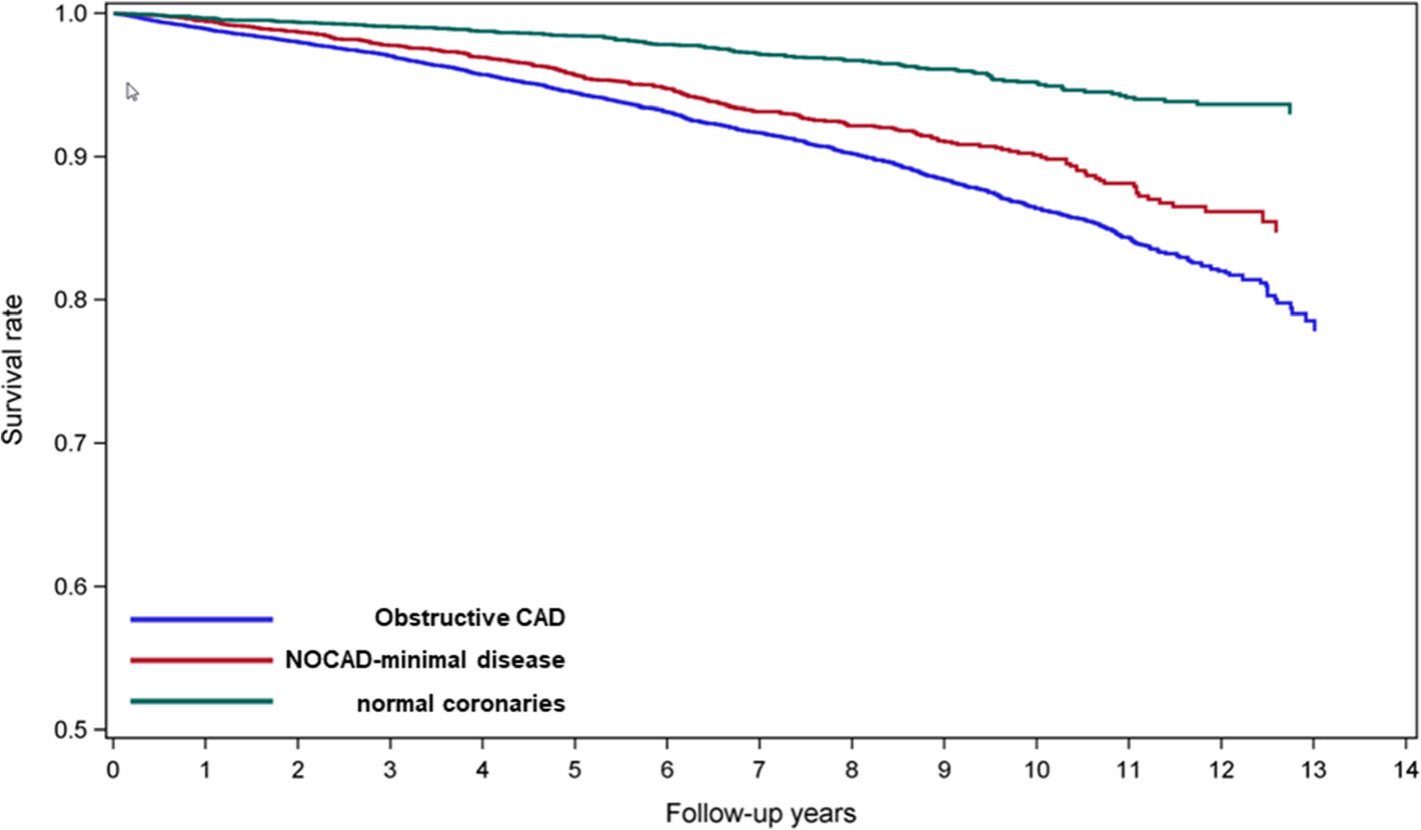
Kaplan Meier curve of patients with stable angina and NOCAD (normal, minimal), and obstructive CAD adjusted for age, hypertension, and diabetes

The crude all-cause mortality at 10 years occurred in 5.3% and 9.8% of stable patients with NOCAD and CAD, respectively (*p* < 0.001). SA patients with NOCAD had a lower risk of all-cause mortality than patients with CAD after adjustment for basic risk factors (Table 2). However, when the NOCAD population was divided in patients with completely normal coronary arteries and patients with minimal disease, the latter appeared to have a similar risk as CAD patients (Table 2). Patients with completely normal coronary arteries had a lower risk compared to patients with CAD. The Kaplan Meier survival curves between NOCAD (normal, minimal), and obstructive CAD adjusted for age, hypertension, and diabetes are depicted in Fig. 2.

**Table 2:** All-cause mortality and adjusted hazard ratios for comparison of CAD patients with NOCAD patients

				Age, DM, HTN- adjusted	
	Deaths n	Total n	10-year rate (%)	HR (95% CI)	*p*-value
NOCAD	398	7478	5.3	1.33 (1.19–1.49)	< 0.001^*^
normal	132	3691	3.6	1.63 (1.36–1.94)	< 0.001^†^
minimal	266	3787	7	1.08 (0.95–1.23)	0.06^‡^
CAD (> 50%)	1068	10,906	9.8	–	–

*: CAD vs. NOCAD; †: CAD vs. normal; ‡: CAD vs. minimal

When NOCAD patients presented with an ACS rather than with SA, they had a 41% increase in mortality risk (NOCAD ACS vs. SA HR 1.41 (1.25–1.6), *p* < 0.001).

### Secondary endpoints in NOCAD SA population

Over a median of 6.5 years of follow up, the percentage of subjects with a repeat catheterization was low (*n* = 543 (7.3%)). Simultaneously, progression to obstructive CAD in patients with NOCAD was small (*n* = 170 (2.3%)), as well as the necessity to perform a PCI (*n* = 128 (1.7%)). Overall, patients with a NOCAD had significantly lower rates of cardiac procedures than patients with stable one-vessel CAD (*p* < 0.001).

### Independent predictors of primary and secondary endpoints

To determine independent predictors of all-cause mortality in the NOCAD population, we performed a multivariate Cox regression analysis, including pre-specified variables as described in the methods’ section. Age above 55 years was the strongest independent predictor of all-cause death (Table 3). Subjects with minimal disease compared with normal coronary arteries were at increased risk of death, and this was higher than the risk associated with the presence of diabetes or smoking (Table 3).

**Table 3:** Independent predictors for all-cause mortality in NOCAD SA patients

Variables	HR (95% CI)	*p*-value
Minimal CAD vs. Normal	1.69 (1.35–2.12)	< 0.001
Age ≥ 55 years vs. Age < 55 years	3.34 (2.48–4.51)	< 0.001
Diabetes mellitus vs. No diabetes	1.5 (1.16–1.94)	0.002
Normal ECG vs. Abnormal	0.67 (0.52–0.87)	0.002
Hypertension vs. No hypertension	1.23 (0.98–1.55)	0.08
Smoker vs. Non-smoker	1.53 (1.23–1.91)	< 0.001
Men vs. Women	1.26 (1.01–1.56)	0.04

Table 4 shows that for the secondary endpoints of future development of obstructive CAD, repeat angiogram, and future PCI, presence of minimal CAD was the strongest independent predictor in all of the three subgroups, followed by DM, and male sex.

**Table 4:** Independent predictors for repeat angiogram, future PCI, and progression of CAD in NOCAD SA patients

	Repeat angiogram (n = 543)		Future PCI (n = 128)		Future CAD (n = 170)	
Variables	HR (95% CI)	*p*-value	HR (95% CI)	*p*-value	HR (95% CI)	*p*-value
Minimal vs. normal	1.87 (1.53–2.29)	< 0.001	3.78 (2.38–6.01)	< 0.001	3.83 (2.59–5.66)	< 0.001
Age ≥ 55 years vs. age < 55 years	1.41 (1.14–1.75)	0.001	1.15 (0.76–1.75)	0.51	1.52 (1.05–2.2)	0.03
Diabetes mellitus vs. no diabetes	1.64 (1.28–2.1)	< 0.001	3.3 (2.15–5.08)	< 0.001	2.03 (1.34–3.07)	0.001
Normal ECG vs. abnormal	0.8 (0.63–1.01)	0.06	1.13 (0.73–1.75)	0.57	0.61 (0.39–0.94)	0.03
Hypertension vs. no hypertension	1.2 (0.98–1.47)	0.07	1.18 (0.78–1.78)	0.44	1 (0.71–1.4)	0.98
Smoker vs. non-smoker	1.15 (0.94–1.4)	0.18	1.05 (0.7–1.57)	0.82	1.42 (1–2.01)	0.05
Men vs. women	1.42 (1.17–1.74)	< 0.001	2.33 (1.52–3.57)	< 0.001	1.78 (1.25–2.52)	0.001

## Discussion

Our large, contemporary study of SA patients with NOCAD and normal LV-function found several key findings. Patients with NOCAD had favourable long-term rates of repeat angiography, future CAD, and PCI. Nevertheless, patients with minimal disease had a similar risk of all-cause mortality as patients with stable CAD. Finally, NOCAD patients presenting with an ACS, had a 41% increase in all-cause mortality compared with those with a SA presentation.

We were able to demonstrate low rates of repeat angiography for patients with NOCAD. Our rate of repeat catheterization is in line with results reported by others [9, 14]. But this is in contrast to reports from the WISE study cohort, which reported higher rates of repeat angiography of 18–34.5% [15, 16]. The WISE cohort had a high proportion of persistently symptomatic women [16]. Data on persistence of symptoms is unfortunately not available to us. We can only speculate whether our lower rate of repeat catheterization is due to lower rate of persistent symptoms, or an increased awareness of physicians of cardiac syndrome X, and microvascular angina. Further potential explanations might be differences in national/regional practices.

We further showed that NOCAD patients presenting with ACS are at increased risk for all-cause mortality. In daily practice however, the finding of NOCAD on coronary angiography is often regarded as insignificant, even when patients present with an ACS. The cause of ACS in NOCAD patients is often unclear, and a variety of etiologies can account for the findings [12, 17]. It is also well documented that a large number of plaque ruptures occur at the site of non-obstructive lesions that lead to thrombotic occlusion [18, 19]. Some of the ACS patients likely had a plaque rupture but the thrombus was not visible at time of cardiac catheterization anymore. Furthermore, plaque erosion is a potential cause for ACS, which might have been missed in some cases [20]. Since we did not routinely perform intravascular ultrasound or optical coherence tomography (OCT), we do not know the percentage of patients with a plaque rupture or erosion without obvious filling defect. Furthermore, it is known that in symptomatic patients with NOCAD, endothelial dysfunction can lead to signs and symptoms of coronary ischemia and is associated with a worse prognosis [9, 21–23]. We do not have any measures of endothelial function in this study. We can only speculate that coronary endothelial dysfunction led to symptoms and was possibly more pronounced in patients presenting with an ACS, which in turn led to a worse outcome. On the other hand, myocarditis and Takotsubo cardiomyopathy are also potential causes for an ACS- like presentation in patients with NOCAD. Albeit Takotsubo was first described in 1990 in a Japanese publication and has gained worldwide recognition since [24], awareness for Takotsubo was delayed in Western countries and it could well be that some of the earlier inclusions in the APPROACH cohort underdiagnosed Takotsubo. Taken together, patients with NOCAD and ACS likely constitute different etiologies [12, 17]. Given that this patient cohort has a 41% increase in risk, a thorough work-up is warranted to better define the etiology in the individual patient, and to appropriately treat patients according to etiology. In clinical reality, patients with an ACS and NOCAD, and no obvious filling defect, or spasm, often receive less secondary preventive measures [5, 17].

In regard to the increased all-cause mortality risk of patients with minimal disease, our data reinforces results from contemporary studies done by coronary computed tomography angiography (CCTA), which showed that patients with NOCAD had a similar mortality risk as patients with obstructive 1-vessel CAD [25]. Concomitantly, the study confirms a graded increase in risk from normal coronaries to non-obstructive disease to obstructive CAD as reported by angiographic studies, as well as by CCTA studies [4, 5, 15, 25–27]. Recently, Maddox et al. were able to show a graded increase of all-cause mortality and MI rate at 1 year from non-obstructive to obstructive CAD in a large, mostly male, cohort of US veterans who presented for an elective coronary angiogram [5]. Although we did not divide the groups by extent of disease, we were able to confirm an increase in all-cause mortality from normal to coronary sclerosis to obstructive disease in an all-comer population for a longer follow-up period. One of the most important findings of our studies was that patients with minimal disease had a similar HR for all-cause mortality as patients with stable CAD. Minimal disease was an independent predictor of similar strength as DM and smoking in our study. This is in line with several other studies. Recent meta-analyses also confirmed a poorer prognosis of patients with minimal disease compared to patients with normal arteries [28]. Furthermore, Lin et al. demonstrated that the detection of NOCAD improves prediction of mortality beyond conventional risk factor assessment [26].

Overall, data appears to accumulate that the finding of NOCAD is not benign and should prompt consideration of secondary prevention measures as used in subjects with stable obstructive CAD. Large-scale studies are warranted to determine the benefit of such measures.

### Limitations

Some limitations apply to this study. Foremost, this was not a randomized controlled trial but a population-based registry study with all its ensued limitations of potential confounding and unmeasured covariates. However, the strength of the APPROACH registry is that it provides a real-world scenario and it captures all deaths and revascularization procedures within the province.

Of note is that APPROACH is a procedure-based registry and not a clinical registry. We cannot exclude a diagnosis, angiogram and/or hospitalization referral bias. However, based on information from an APPROACH ACS registry, it is known that 73.9% of women and 85.1% of men (*p* < 0.05) who present to our region with ACS undergo catheterization. Furthermore, we used multivariable adjustment as strategy to account for baseline differences between patients with NOCAD and CAD. Nevertheless, residual confounding cannot be excluded.

One of the major limitations is that there is no data on the cause of death or cardiovascular death in particular in the APPROACH database. Rehospitalisation rates for ischemia or heart failure, stroke, or quality of life measures are not known for NOCAD patients. Those endpoints have been proven to be of special importance in patients with NOCAD [4, 14, 16]. Nonetheless, data on the hard endpoint of all-cause mortality is robust and of clinical significance. We are also lacking data on long-term medication use or risk factor control. During the long follow-up period from 1995 to 2012, medical management of patients also might have changed. For instance, high dose statin therapy became more common in the early twenty-first century. Also, physicians might have been more prone to use secondary preventive measures in NOCAD over time. A further concern is that it can be difficult to discern normal coronaries from NOCAD. Intravascular ultrasound data, or OCT would have been beneficial but is rarely used for diagnostic angiograms under the specified conditions. Furthermore, grade of stenosis was not assessed in a core lab by quantitative coronary analysis. Therefore, under- or overestimation of stenosis grade cannot be ruled out. In spite of these limitations, our study represents a real-world scenario and is similar to other large-scale registries.

In regard to the study design, one of the main drawbacks is the lack of an asymptomatic, normal control. However, previous studies have shown that there is a graded increase from asymptomatic controls to symptomatic with normal coronary arteries [4].

## Conclusion

In conclusion, stable patients with NOCAD have low rates of repeat angiography, future CAD, and PCI. This is reassuring when dealing with and treating these patients. However, subjects with minimal disease should be considered at similar risk as patients with stable, obstructive CAD, which might argue for more aggressive risk factor control in these patients. Also, NOCAD patients presenting with an ACS have a 41% increase in risk for all-cause mortality which might warrant more intensive diagnostic evaluation, treatment, and follow-up.

## Additional file

Additional file 1: **Table S1.** Comparison of Baseline Characteristics of Stable Angina Patients with NOCAD: normal coronaries vs. minimal disease. (DOCX 15 kb)

## Abbreviations

ACS: acute coronary syndrome
APPROACH: Alberta Provincial Project for Outcome Assessment in Coronary Heart Disease
CABG: coronary artery bypass grafting
CAD: coronary artery disease
DM: diabetes mellitus
EF: ejection fraction
HR: hazard ratio
HTN: hypertension
NOCAD: non-obstructive coronary artery disease
OCT: optical coherence tomography
PCI: percutaneous coronary intervention
SA: stable angina
