# Supplementary material for: Impact of clinical presentation and presence of coronary sclerosis on long-term outcome of patients with non-obstructive coronary artery disease
Source: BMC Cardiovasc Disord. 2018 Aug 22;18:173. doi: 10.1186/s12872-018-0908-z (PMC6106760; doi:10.1186/s12872-018-0908-z)
Supplement: Supplementary file 1 — Table S1. Comparison of Baseline Characteristics of Stable Angina Patients with NOCAD: normal coronaries vs. minimal disease. (DOCX 15 kb) [file 12872_2018_908_MOESM1_ESM.docx]

**Additional file 1**

**Table S1: Comparison of Baseline Characteristics of Stable Angina Patients with NOCAD:  normal coronaries vs. minimal disease.**

|  | **Normal coronaries** **(n=3691)** | **Minimal disease**  **(n=3787)** | **p-value*** |
| --- | --- | --- | --- |
| Age, mean years | 56.0±10.7 | 61.5.2±10.4 | <0.0001 |
| Female (%) | 1954 (52.9%) | 1563 (41.3%) | <0.0001 |
| EF calculated (%) | 64.6±7.1 (n= 1220) | 65.2±7.8 (n= 1379) | 0.09 |
| **Cardiovascular risk factors:** | | | |
| Hypertension (%) | 54.9 | 65.7 | <0.0001 |
| Dyslipidemia (%) | 61.9 | 69.3 | <0.0001 |
| Diabetes mellitus (%) | 14.5 | 19.0 | <0.0001 |
| Smoker-current/ previous (%) | 48.7 | 56.3 | <0.0001 |
| Current smoker (%) | 16.9 | 18.4 | 0.08 |
| Positive family history (%) | 28.8 | 30.4 | 0.13 |
| **Medications at time of cath:** | | |  |
| Aspirin | 2551/3524 (72.4%) | 2435/3635 (75.2%) | 0.006 |
| P2Y12 Inhibitor | 210/3364 (6.2%) | 270/3514 (7.7%) | 0.02 |
| Beta-blockers | 1733/3471 (49.9%) | 1828/3589 (50.9%) | 0.40 |
| Statins | 1437/3379 (42.5%) | 1848/3547 (52.1%) | <0.0001 |
| Calcium channel blockers | 592/3370 (17.6%) | 677/3541 (19.1%) | 0.1 |
| ACE-inhibitor | 828/3404 (24.3%) | 1129/ 3565 (31.7%) | <0.0001 |
| Long acting nitrates | 406/3355 (12.1%) | 465/3507 (13.1%) | 0.19 |
| Insulin | 83/2743 (3.0%) | 110/3017 (3.6%) | 0.19 |

*for comparison minimal vs. normal
